# From the Cochrane Library: Leukotriene Receptor Antagonists for Eczema

**DOI:** 10.2196/50434

**Published:** 2024-04-12

**Authors:** Lauren Marie Toledo, Ramiro Rodriguez, Torunn E Sivesind, Efstratios Vakirlis, Reiji Kojima, Robert P Dellavalle

**Affiliations:** 1 Lake Erie College of Osteopathic Medicine Bradenton, FL United States; 2 University of Colorado Anschutz Medical Campus Aurora, CO United States; 3 Department of Dermatology and Venereology School of Medicine Aristotle University of Thessaloniki Thessaloniki Greece; 4 Department of Health Sciences School of Medicine University of Yamanashi Yamanashi Japan; 5 University of Colorado School of Medicine Aurora, CO United States; 6 Rocky Mountain Regional VA Medical Center Aurora, CO United States

**Keywords:** eczema, atopic dermatitis, leukotriene receptor antagonists, systematic reviews, dermatitis, inflammatory, skin disease, skin, clinical, medications, management, receptor, antagonist

## Introduction

Atopic dermatitis (AD), a chronic inflammatory skin disease, is estimated to affect up to 10% of adults and 20% of children worldwide [1]. Clinical manifestations include pruritus, skin lesions, and dry scaly skin [2,3]. First-line treatment includes topical steroids and emollients, with systemic steroids or immune modulators for moderate-to-severe AD. Despite the standard practice of using topical corticosteroids in AD treatment, long-term use poses the risk of local adverse effects of skin thinning, striae, and purpura, or systemic effects such as growth suppression and suppression of the hypothalamic-pituitary axis [4]. Other medications, such as leukotriene receptor antagonists (LTRAs), are being researched as an alternative treatment option [5]. A 2018 Cochrane review, “Leukotriene receptor antagonists for eczema” [6], examined clinical trials to determine if there is sufficient evidence to recommend LTRAs for use in patients with AD but concluded that there was limited, low-quality evidence of its efficacy and safety.

## Methods

This Cochrane review extracted data across 5 studies and 202 participants to evaluate the evidence of LTRA effectiveness in AD. Of these studies, 3 assessed the efficacy of LTRAs compared to a placebo and 2 assessed the effectiveness of LTRAs versus conventional treatment (combined antihistamines and topical steroids). All assessed the effectiveness of the LTRA montelukast, met inclusion criteria of being a randomized controlled trial (RCT) and assessing patients with moderate-to-severe eczema, and tested interventions for the acute or chronic phase of AD. Interventions assessed independent administration of montelukast (oral or intravenous) or montelukast in combination with other topical and systemic treatments (corticosteroids, topical calcineurin inhibitors, immunomodulators, or placebo).

## Results

Only 1 RCT resulted in greater improvement with LTRA intervention compared to conventional treatment but was of low quality. None of the studies addressed long-term control (primary outcome) or higher quality of life and lower emollient requirement (secondary outcomes) at all (Table 1). The quality of supporting evidence was assessed by GRADE (Grading of Recommendations, Assessment, Development, and Evaluations) based on 5 domains: limitations and risk of bias, inconsistency, direct relation of evidence, imprecision, and publication bias. While valuing the certainty of evidence, it is essential to be aware that judgments may vary between individuals with this method. The authors note it is challenging to draw firm conclusions from the results of these studies because the studies had an unclear or high risk of bias, including but not limited to selection and detection. Limitations of the studies included the absence of testing other LTRAs besides montelukast, inclusion of only adult participants and participants with moderate-to-severe eczema, and a small sample size. Detection biases were present in 2 studies due to the lack of blinding of the outcome assessment; performance bias was of concern in 1 study due to the lack of blinding of participants and personnel in an open RCT. Potential confounders (eg, diet, detergent, household chemicals, climate, location, allergens) were not assessed, which could contribute to an underestimate or overestimate of the true association between LTRAs and AD.

**Table 1:** Summary of randomized controlled trials assessed in the Cochrane review, “Leukotriene receptor antagonists for eczema” [6].

	Study (year)				
	Capella et al (2001)	Friedmann et al (2007)	Nettis et al (2002)	Rahman et al (2006)	Veien et al (2005)
Study title	A randomized trial of leukotriene receptor antagonist montelukast in moderate‐to-severe atopic dermatitis of adults	A double‐blind, placebo‐controlled trial of montelukast in adult atopic eczema	Efficacy and tolerability of montelukast as a therapeutic agent for severe atopic dermatitis in adults	Effectiveness of montelukast in the treatment of atopic dermatitis	Montelukast treatment of moderate to severe atopic dermatitis in adults: a randomized, double‐blind, placebo‐controlled trial
Participants, N	32	58	20	31	53
Type of trial	Single blind	Double blind	Double blind	Open label	Double blind
Length of study	6 weeks	8 weeks	6 weeks	4 weeks	4 weeks
Intervention vs comparator	Oral montelukast + oral placebo + topical placebo gel vs (conventional) oral cetirizine + oral clarithromycin + topical steroid creams	Montelukast vs placebo	Montelukast vs placebo	Montelukast vs (conventional) antihistamine + 1% topical hydrocortisone	Montelukast vs placebo
Montelukast dose	10 mg for adults, 5 mg for children	10 mg for adults, 5 mg for children	10 mg for adults, 5 mg for children	10 mg for adults, 5 mg for children	10 mg for adults, 5 mg for children
Scale	SCORAD^a^	SASSAD^b^	SCORAD	SCORAD	Modified EASI^c^
Study conclusions	Significant improvement in SCORAD scores of both montelukast and placebo groups but no significant difference	No significant difference between montelukast and placebo for pruritus improvement	20% significant reduction in SCORAD with montelukast. Montelukast was superior	Significant improvement in SCORAD with montelukast compared to conventional treatment. Montelukast was superior	No significant difference between the EASI scores of montelukast and placebo groups
Reason for lack of evidence	Low quality of evidence, small sample size, high risk of bias	Low quality of evidence, small sample size	Low quality of evidence, small sample size	Low quality of evidence, small sample size	Low quality of evidence, small sample size, high risk of bias
Standard mean difference (95% CI), inverse variance, random	Not provided	–0.03 (–0.54 to 0.49)	1.09 (0.13 to 2.04)	10.57 (4.58 to 16.56)	0.20 (–0.34 to 0.74)
Adverse effects	None	Dizziness reported; mild in nature except for a brief septicemic illness	None	None	None

^a^SCORAD: Scoring Atopic Dermatitis.

^b^SASSAD: Six Area, Six Sign Atopic Dermatitis.

^c^EASI: Eczema Area and Severity Index.

## Discussion

Experimental data on the involvement of leukotrienes in allergic inflammation suggests LTRA therapy might be promising for the treatment of AD [3]; however, the results to date are unclear and lack uniformity. The increasing incidence of AD highlights the need for additional investigation to identify the most effective treatments, especially those that can be used as long-term maintenance therapy. While there is no compelling evidence in this review for or against LTRA use for AD treatment, a large, well-designed RCT with multiple LTRAs would help better understand LTRA’s role in long-term AD management.

## Abbreviations

AD: atopic dermatitis
GRADE: Grading of Recommendations, Assessment, Development, and Evaluations
LTRA: leukotriene receptor antagonist
RCT: randomized controlled trial
